# Reverse engineering of a Hamiltonian by designing the evolution operators

**DOI:** 10.1038/srep30151

**Published:** 2016-07-22

**Authors:** Yi-Hao Kang, Ye-Hong Chen, Qi-Cheng Wu, Bi-Hua Huang, Yan Xia, Jie Song

**Affiliations:** 1 Department of Physics, Fuzhou University, Fuzhou 350002, China; 2 Department of Physics, Harbin Institute of Technology, Harbin 150001, China

## Abstract

We propose an effective and flexible scheme for reverse engineering of a Hamiltonian by designing the evolution operators to eliminate the terms of Hamiltonian which are hard to be realized in practice. Different from transitionless quantum driving (TQD), the present scheme is focus on only one or parts of moving states in a *D*-dimension (*D* ≥ 3) system. The numerical simulation shows that the present scheme not only contains the results of TQD, but also has more free parameters, which make this scheme more flexible. An example is given by using this scheme to realize the population transfer for a Rydberg atom. The influences of various decoherence processes are discussed by numerical simulation and the result shows that the scheme is fast and robust against the decoherence and operational imperfection. Therefore, this scheme may be used to construct a Hamiltonian which can be realized in experiments.

Executing computation and communication tasks^1^,^2^,^3^,^4^ with time-dependent interactions in quantum information processing (QIP)^5^,^6^,^7^,^8^,^9^,^10^,^11^,^12^ have attracted more and more interests in recent years. It has been shown that, the adiabatic passage, resonant pulses, and some other methods can be used to realize the evolution process. Among of them, the adiabatic passage techniques are known for their robustness against variations of experimental parameters. Therefore, many schemes have been proposed with adiabatic passage techniques in quantum information processing field. For example, rapid adiabatic passage, stimulated Raman adiabatic passage, and their variants^13^,^14^,^15^,^16^,^17^,^18^,^19^,^20^,^21^,^22^ have been widely used to perform population transfers in two- or three-level systems. The system keeps in the instantaneous ground state of its time-dependent Hamiltonian during the entire evolution process under an adiabatic control of a quantum system. To ensure that the adiabatic condition is always satisfied, the control parameters in the Hamiltonian should be well designed, which usually issue in relatively long execution time. Although little heating or friction will be created when the system remains in the instantaneous ground state, the long time required may make the operation useless or even impossible to implement because decoherence would spoil the intended dynamics. On the other hand, using resonant pulses, the scheme may has a relatively high speed, but it requires exact pulse areas and resonances. Therefore, accelerating the adiabatic passage towards the perfect final outcome is a good idea and perhaps the most reasonable way to actually fight against the decoherence that is accumulated during a long operation time. Consequently, some alternative approaches have been put forward by combining the virtues of adiabatic techniques and resonant pulses together for achieving controlled quantum state evolutions with both high speed and fidelity, such as optimal control theory^23^,^24^,^25^ and composite pulses^26^,^27^. Recently, by designing nonadiabatic shortcuts to speed up quantum adiabatic process, a new technique named “shortcuts to adiabaticity” (STA)^28^,^29^,^30^,^31^,^32^,^33^,^34^,^35^,^36^,^37^,^38^,^39^ opens a new chapter in the fast and robust quantum state control. As two famous methods of STA, “Transitionless quantum driving” (TQD)^31^,^32^,^33^,^34^ and inverse engineering^34^,^35^,^36^,^37^,^38^ based on Lewis-Riesenfeld invariants^40^ have been intensively focused, They have been applied in different kinds of fields including “fast quantum information processing”, “fast cold-atom”, “fast ion transport”, “fast wave-packet splitting”, “fast expansion”, etc.^41^,^42^,^43^,^44^,^45^,^46^,^47^,^48^,^49^,^50^,^51^,^52^,^53^,^54^,^55^,^56^,^57^,^58^,^59^,^60^. For example, with invariant-based inverse engineering, a fast population transfer in a three-level system has been achieved by Chen and Muga^56^. Chen *et al*.^57^ have proposed a scheme for fast generation of three-atom singlet states by TQD. These schemes have shown the powerful application for invariant-based inverse engineering and TQD in QIP.

It has been pointed out in ref. 34 that, invariant-based inverse engineering and TQD are strongly related and potentially equivalent to each other. Invariant-based method is convenience and effective with a Hamiltonian which admits known structures for the invariants. But for most systems, the invariants are unknown or hard to be solved. As for TQD, it will not meet this difficult point. However, some terms of Hamiltonian constructed by TQD, which are difficult to be realized in experiments, may appear when we accelerate adiabatic schemes. Therefore, how to avoid these problematic terms is a notable problem. Till now, some schemes^61^,^62^,^63^,^64^,^65^,^66^,^67^,^68^,^69^,^70^ have been proposed to solve the problem of the TQD method recently. For example, Ibáñez *et al*.^64^ have produced a sequence of STA by examining the limitations and capabilities of superadiabatic iterations. Ibáñez *et al*.^65^ have also studied the STA for a two-level system with multiple Schrödinger pictures, and subsequently, Song *et al*.^66^ have expanded the method in a three-level system based on two nitrogen-vacancy-center ensembles coupled to a transmission line resonator. Moreover, without directly using the counterdiabatic Hamiltonian, Torrontegui *et al*.^67^ have used the dynamical symmetry of the Hamiltonian to find alternative Hamiltonians that achieved the same goals as speed-up schemes with Lie transforms. Chen *et al*.^70^ have proposed a method for constructing shortcuts to adiabaticity by a substitute of counterdiabatic driving terms.

In this paper, inspired by TQD and the previous schemes^61^,^62^,^63^,^64^,^65^,^66^,^67^,^68^,^69^,^70^, a new scheme for reverse engineering of a Hamiltonian by designing the evolution operators is proposed for eliminating the terms of Hamiltonian which are hard to be realized in practice. The present scheme is focus on only one or parts of moving states in a *D*-dimension (*D* ≥ 3) system, that is different from TQD with which all instantaneous eigenstates evolve parallel. According to the numerical simulation, the present scheme not only contains the results of TQD, but also has more free parameters, which make this scheme more flexible. Moreover, the problematic terms of Hamiltonian may be eliminated by suitably choosing these new free parameters. For the sake of clearness, an example is given to realize the population transfer for a Rydberg atom, where numerical simulation shows the scheme is effective. Therefore, this scheme may be used to construct a Hamiltonian which can be realized in experiments.

The article is organized as follows. In the section of “Reverse engineering of a Hamiltonian”, we will introduce the basic principle of the scheme for reverse engineering of a Hamiltonian by designing the evolution operators. In the section of “The population transfer for a Rydberg atom”, we will show an example using the present scheme to realize the population transfer for a Rydberg atom. Finally, conclusions will be given in the section of “Conclusion”.

## Reverse engineering of a Hamiltonian

We begin to introduce the basic method of the scheme for reverse engineering of a Hamiltonian by designing the evolution operators. Firstly, we suppose that the system evolves along the state |*ϕ*_1_(*t*)〉 and the initial state of the system is |*ψ*(0)〉. So, the condition |*ϕ*_1_(0)〉 = |*ψ*(0)〉 should be satisfied. We can obtain a complete orthogonal basis {|*ϕ*_*n*_(*t*)〉} through a process of completion and orthogonalization. Therefore, the vectors in basis {|*ϕ*_*n*_(*t*)〉} satisfy the orthogonality condition 〈*ϕ*_*m*_(*t*)|*ϕ*_*n*_(*t*)〉 = *δ*_*mn*_ and the completeness condition 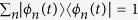. Since the system evolves along |*ϕ*_1_(*t*)〉, the evolution operator can be designed as


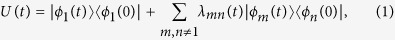


where parameters *λ*_*mn*_(*t*) (*m, n* ≠ 1) are chosen to satisfy the unitary condition *UU*^†^ = *U*^†^*U* = 1. Submitting the unitary condition into Eq. (1), we obtain


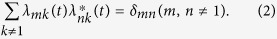


Secondly, according to Schrödinger equation (ħ = 1), we have


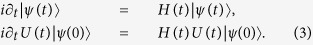


On account of the arbitrariness of |*ψ*(0)〉, Eq. (3) can be written by


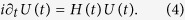


The Hamiltonian can be formally solved from Eq. (4), and be given as


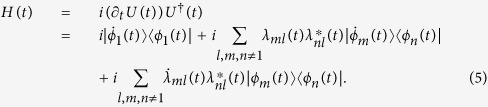


By submitting Eq. (2) into Eq. (5), the Hamiltonian in Eq. (5) can be described as


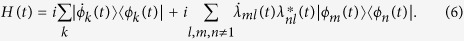


Different from TQD, which gives Hamiltonian in the following from


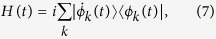


the present scheme has more free parameters *λ*_*mn*_(*t*). Therefore, this scheme may construct some new and different Hamiltonians. Moreover, when parameters *λ*_*mn*_ (*m, n* ≠ 1) are independent of time, Eq. (6) will degenerate into Eq. (7), which shows that the present scheme contains the results of TQD. On the other hand, once the unitary condition *UU*^†^ = *U*^†^*U* = 1 for evolution operator is satisfied, the Hamiltonian given in Eq. (6) should be a Hermitian operator, because


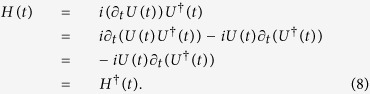


As an extension, for a *N*-dimension system (*N* ≥ 4), the evolution operator can be designed as


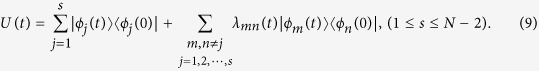


Then, the initial state |*ψ*(0)〉 of the system can be expressed by the superposition of {|*ϕ*_*j*_(0)〉} (*j* = 1, 2, …, *s*). Thus, the system can evolve along more than one moving states in this case. This might sometimes help us to simplify the design of the system’s Hamiltonian.

## The population transfer for a Rydberg atom

**Figure 1:**
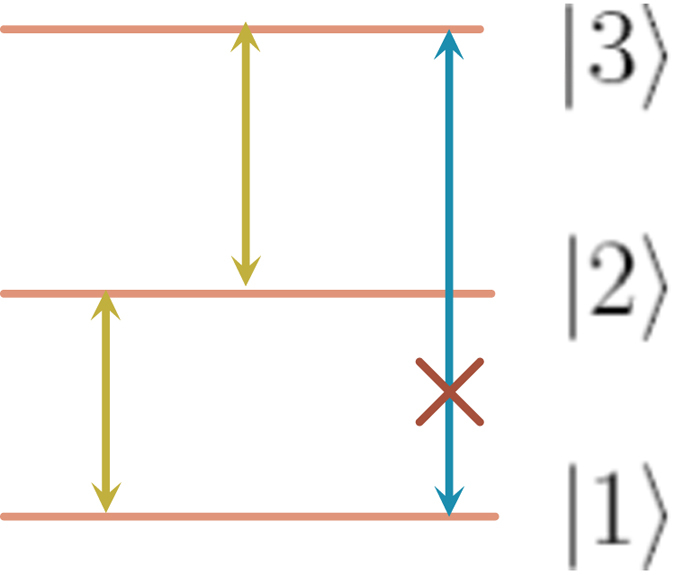
Energy levels of the three-energy-level Rydberg atom.

For the sake of clearness, we give an example to emphasize the advantages of the scheme. Here, we consider a Rydberg atom with the energy levels shown in Fig. 1. The transition between |1〉 and |3〉 is hard to realize. So, the Hamiltonian of the Rydberg atom is usually written as the following form


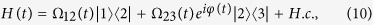


where, Ω_12_ and Ω_23_ are the Rabi frequencies of laser pulses, which drive the transitions |1〉 ↔ |2〉 and |2〉 ↔ |3〉, respectively, and they are *φ*-dephased from each other. Suppose the initial state of the three-energy-level Rydberg atom is |1〉, the target state is |Ψ_*tar*_〉 = cos *μ*|1〉 + sin *μ*|3〉. We choose a complete orthogonal basis as below


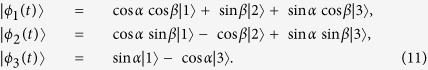


With the unitary condition in Eq. (2), the evolution operator can take this form


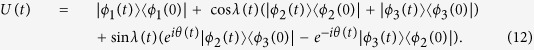


According to Eq. (6), the evolution operator in Eq. (12) gives the following Hamiltonian


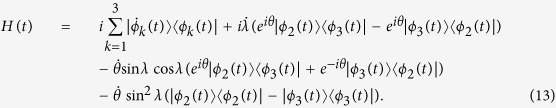


For simplicity, we set *θ* = 0 here, the Hamiltonian in Eq. (13) can be written by


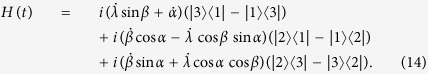


Here, the Hamiltonian in Eq. (14) is already a Hermitian operator. To eliminate the terms with |1〉 〈3| and |3〉 〈1|, which are difficult to realize for the three-energy-level Rydberg atom, we set 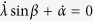. Eq. (14) will be changed into


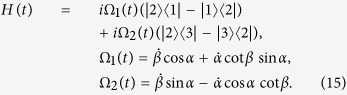


For simplicity, we suppose the initial time is *t*_*i*_ = 0 and the final time is *t*_*f*_ = *T*, so *T* is the total interaction time. To satisfy the boundary conditions *α*(0) = 0, *α*(*T*) = *μ*, 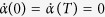, *β*(0) = *β*(*T*) = 0, 
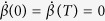
 and avoid the singularity of Hamiltonian, we choose the parameters as


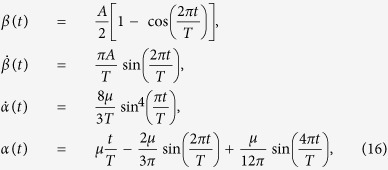


where *A* is an arbitrary constant. Then, the Hamiltonian in Eq. (15) can be written by


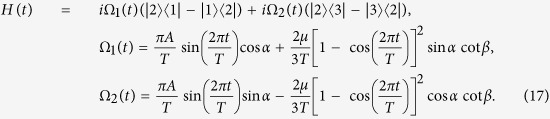


For the sake of obtaining a relatively high speed, the values of Ω_1_*T* and Ω_2_*T* in Eq. (17) should not be too large. Noticing that, with *A* increasing, *πA* increases while cot *β* decreases. Therefore, to obtain a relatively small |Ω_1_*T*| and |Ω_2_*T*|, *A* should be neither too large nor too small. Therefore, we choose *A* = 1 here. However, we can see from Eq. (17) that the functions of Rabi frequencies Ω_1_(*t*) and Ω_2_(*t*) are too complex for experimental realization. Fortunately, we can solve the problem by using simple functions to make a curve fitting for the Ω_1_(*t*) and Ω_2_(*t*). As an example, 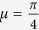 is taken here. We use 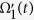 and 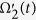 in the following, which are linear superposition of the Gaussian or trigonometric functions, to make a curve fitting for the Ω_1_(*t*) and Ω_2_(*t*),


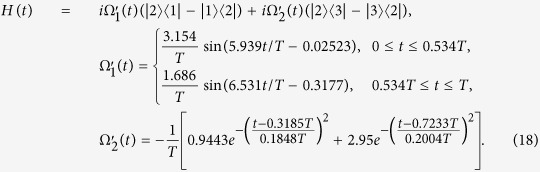


In this case, we have 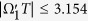 and 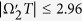.

**Figure 2:**
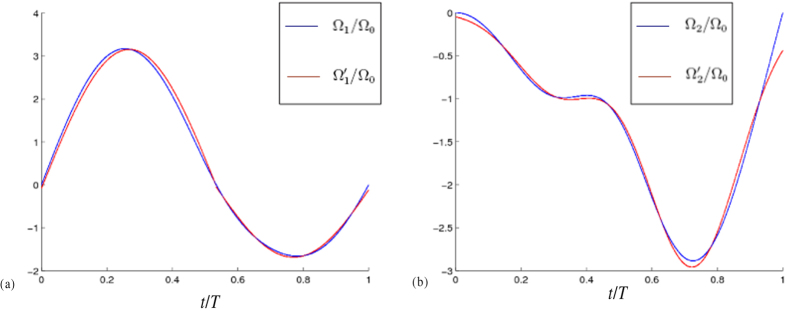
(**a**) Ω1T and 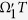 versus *t*/*T* with *μ* = *π*/4. (**b**) Ω_2_*T* and Ω_2′_*T* versus *t*/*T* with *μ* = *π*/4 and *A* = 1.

**Figure 3:**
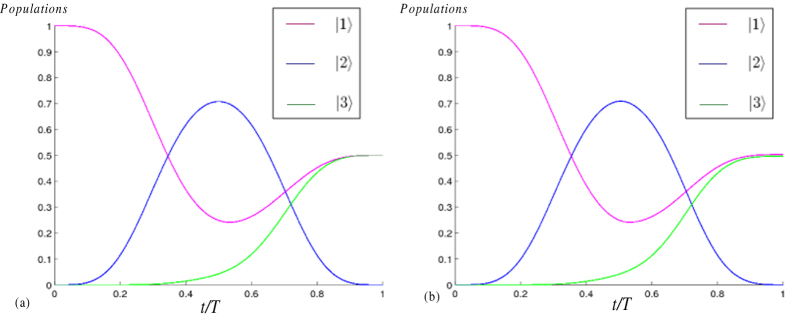
(**a**) Populations of states |1〉, |2〉 and |3〉 versus *t*/*T* when the Rydberg atom is driven by laser pulses with Rabi frequencies Ω_1_ and Ω_2_. (**b**) Populations of states |1〉, |2〉 and |3〉 versus *t*/*T* when the Rydberg atom is driven by laser pulses with Rabi frequencies 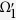 and 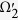. Here we set the parameters *μ* = *π*/4 and *A* = 1.

To compare the values of Ω_1_(*t*) and 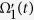, Ω_2_(*t*) and 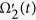, we plot Ω_1_*T* and 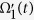 versus *t*/*T* with *μ* = *π*/4 and *A* = 1 in Fig. 2(a) and plot Ω_2_*T* and 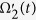 versus *t*/*T* with *μ* = *π*/4 and *A* = 1 in Fig. 2(b). From Fig. 2(a,b), one can find that the curves of Ω_1_(*t*) and 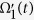 (Ω_2_(*t*) and 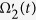) are well matched with each other. Therefore, we may use 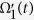 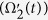 instead of Ω_1_(*t*) (Ω_2_(*t*)) to obtain the same effect. To test the effectiveness of the approximation by using 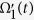 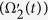 instead of Ω_1_(*t*) (Ω_2_(*t*)), a simulation for the varies of populations of states |1〉, |2〉 and |3〉 when the Rydberg atom is driven by laser pulses with Rabi frequencies Ω_1_(*t*) and Ω_2_(*t*) with parameters *μ* = *π*/4 and *A* = 1, is shown in Fig. 3(a). We can see from Fig. 3(a) that the evolution is consonant with the expectation coming from the evolution operator in Eq. (12). As a comparison, a simulation for the varies of populations of states |1〉, |2〉 and |3〉 when the Rydberg atom is driven by laser pulses with Rabi frequencies Ω′_1_(*t*) and Ω′_2_(*t*) with parameters *μ* = *π*/4 and *A* = 1, is shown in Fig. 3(b). As shown in Fig. 3(a,b), we can conclude that the approximation by using 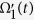 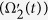 instead of Ω_1_(*t*) (Ω_2_(*t*)) is effective here. In addition, seen from Fig. 3, the population of intermediate state |2〉 reaches a peak value about 0.72, because the system does not evolve along the dark state of the Hamiltonian of the system but a nonadiabatic shortcut, which greatly reduces the total evolution time.

Since most of the parameters are hard to faultlessly achieve in experiment, that require us to investigate the variations in the parameters caused by the experimental imperfection. We would like to discuss the fidelity *F* = |〈Ψ_*tar*_|*ϕ*_1_(*T*)〉|^2^ with the deviations *δT*, 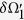 and 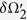 of total interaction time *T*, Rabi frequencies of laser pulses 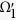 and 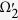 being considered.

**Figure 4:**
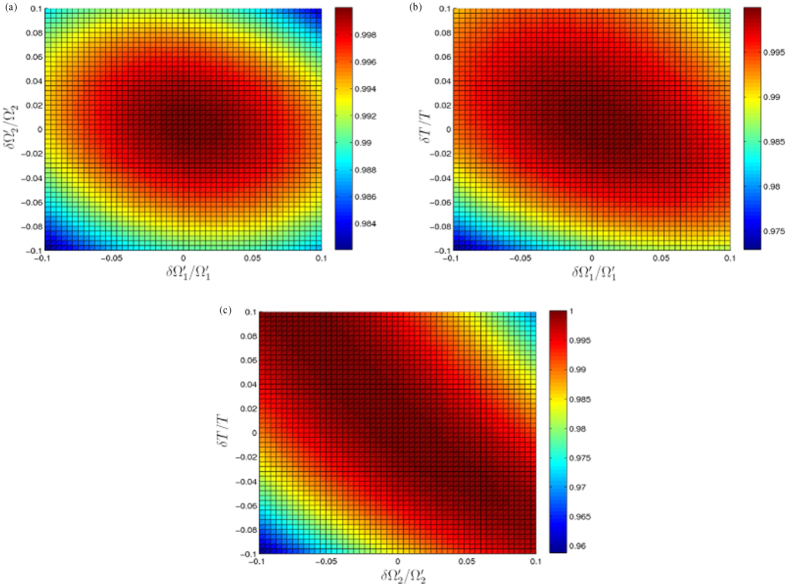
(**a**) Fidelity *F* of the target state versus 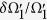 and 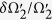. (**b**) Fidelity *F* of the target state versus 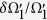 and *δT*/*T*. (**c**) Fidelity *F* of the target state versus 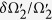 and *δT*/*T*. Here we set the parameters *μ* = *π*/4 and *A* = 1.

Firstly, we plot *F* versus 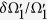 and 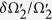 with parameters *μ* = *π*/4 and *A* = 1 in Fig. 4 (a). Moreover, we calculate the exact values of the fidelities *F* at some boundary points of Fig. 4 (a) and show the results in Table 1. According to Table 1 and Fig. 4 (a), we find that the final fidelity *F* is still higher than 0.9822 even when the deviation 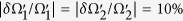. Therefore, the realizing of the population transfer for a Rydberg atom given in this paper is robust against deviations 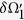 and 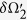 of Rabi frequencies 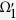 and 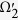 for laser pulses.

Secondly, we plot *F* versus 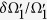 and *δT*/*T* with parameters *μ* = *π*/4 and *A* = 1 in Fig. 4 (b). Moreover, 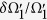 and *δT*/*T* with corresponding fidelity *F* are shown in Table 2. Seen from Table 2 and Fig. 4 (b), we obtain that the fidelity *F* is still high than 0.9729 even when the deviation 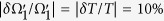. So, the scheme is insensitive to deviations 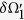 and *δT*.

Thirdly, *F* versus 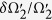 and *δT*/*T* with parameters *μ* = *π*/4 and *A* = 1 is plotted in Fig. 4 (c). And 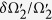 and *δT*/*T* with corresponding fidelity *F* are given in Table 3. As indicated in Table 3 and Fig. 4 (c), the fidelity *F* is still high than 0.9588 even when the deviation 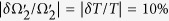. Moreover, when deviations of 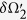 and *δT* have the different signs (one negative and one positive), the fidelity *F* can still keep in a high level. Hence, we can say the scheme suffers little from deviations 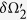 and *δT*.

Fourthly, we discuss the fidelity *F* when 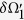, 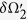 and *δT* are all considered. Some samples are given in Table 4. Table 4 shows that the fidelity *F* is still with a high level when the three deviations 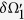, 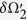 and *δT* are all considered. Moreover, in the worst case, when 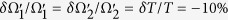, the fidelity *F* is still higher than 0.9469.

According to the analysis above, we summarize that, the scheme to realize the population transfer for a Rydberg atom is robust against operational imperfection.

**Figure 5:**
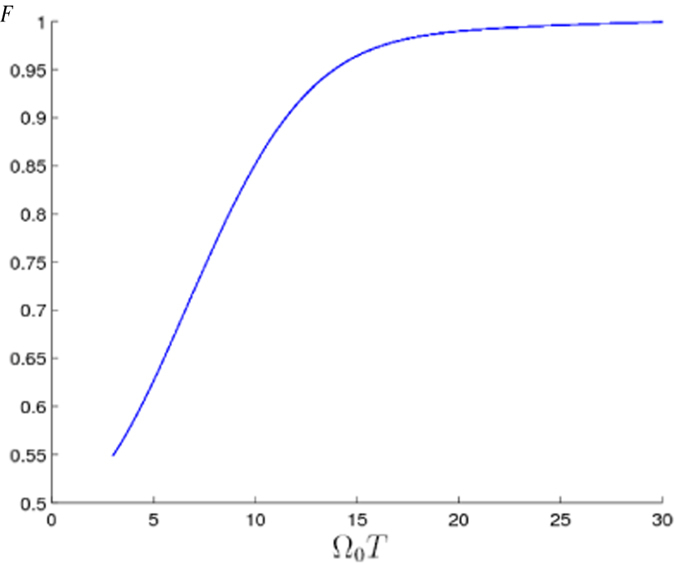
Fidelity *F* of the target state versus Ω_0_*T* with the STIRAP method.

To prove that the present scheme can be used to speed up the system’s evolution and construct the shortcut to adiabatic passages, we make a comparison between the present scheme and the fractional stimulated Raman adiabatic passage (STIRAP) method via dark state 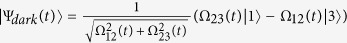 of Hamiltonian shown in Eq. (10). According to STIRAP method, by setting boundary condition


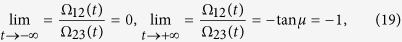


one can design the Rabi frequencies Ω_12_(*t*) and Ω_23_(*t*) as following


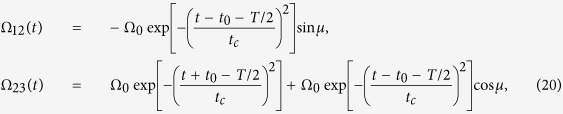


where Ω_0_ denotes the pulse amplitude, *t*_*c*_ and *t*_0_ are some related parameters. Setting *t*_*c*_ = 0.19*t*_*f*_ and *t*_0_ = 0.14*t*_*f*_, Rabi frequencies Ω_12_(*t*) and Ω_23_(*t*) can well satisfy the boundary condition in Eq. (19). We plot Fig. 5 to show the fidelity *F* when the Rydberg atom is driven by laser pulses with Rabi frequencies Ω_12_(*t*) and Ω_23_(*t*) shown in Eq. (20) versus Ω_0_*T*. And a series of samples of Ω_0_*T* and corresponding fidelity *F* are shown in Table 5. From Fig. 5 and Table 5, we can see that, to meet the adiabatic condition and obtain a relatively high fidelity by using STIRAP method, one should take Ω_0_*T* about 30. Moreover, when Ω_0_*T* = 3.154, the adiabatic condition is badly violated and the fidelity is only 0.5538 for STIRAP method. But for the present scheme, we can obtain *F* = 1.000 while 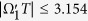 and 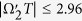. Therefore, the evolution speed with the present scheme is faster a lot comparing with that using STIRAP method. It confirms that the present scheme can be used to speed up the system’s evolution and construct the shortcut to adiabatic passages. Therefore, we conclude that the present scheme can construct a Hamiltonian with both fast evolution process and robustness against operational imperfection.

**Figure 6:**
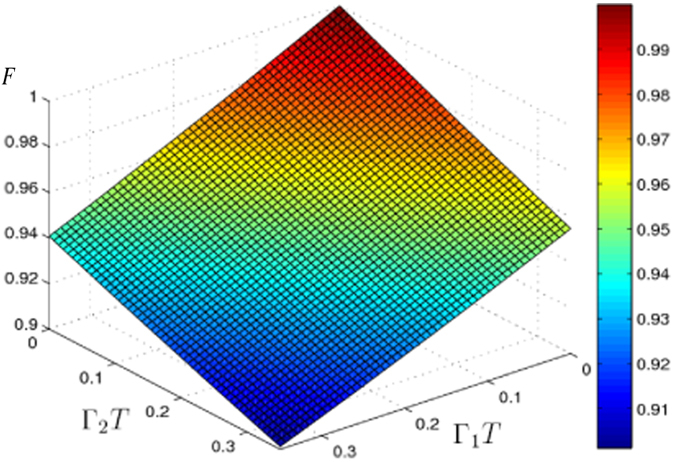
Fidelity *F* of the target state versus Γ_1_/Ω_0_ and Γ_2_/Ω0.

In the end, we discuss the fidelity *F* is robust to the decoherence mechanisms. In this scheme, the atomic spontaneous emission plays the major role. The evolution of the system can be described by a master equation in Lindblad form as following


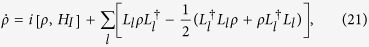


where, *L*_*l*_ is the Lindblad operator. There are two Lindblad operators here. They are 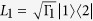 and 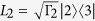, in which, Γ_1_ and Γ_2_ are the atomic spontaneous emission coefficients for |2〉 → |1〉 and |3〉 → |2〉, respectively. Fidelity *F* versus Γ_1_*T* and Γ_2_*T* is plotted in Fig. 6. From Fig. 6, we can see that the fidelity *F* decreases when Γ_1_ and Γ_2_ increase. When in the case of strong coupling 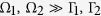, the influence caused by atomic spontaneous emission is little. For example, if Γ_1_ = Γ_2_ = 0.01 × 3.154/*T*, the fidelity is 0.9901. Even when Γ_1_ = Γ_2_ = 0.1 × 3.154/*T*, the fidelity is 0.9101, still higher than 0.9. With current experimental technology, it is easy to obtain a laser pulse with Rabi frequency much larger than the atomic spontaneous emission coefficients. Therefore, the population transfer for a Rydberg atom with the reverse engineering scheme given here can be robustly realized.

## Conclusion

In conclusion, we have proposed an effective and flexible scheme for reverse engineering of a Hamiltonian by designing the evolution operators. Different from TQD, the present scheme is focus on only one or parts of moving states in a *D*-dimension (*D* ≥ 3) system. The numerical simulation has indicated that the present scheme not only contains the results of TQD, but also has more free parameters, which make this scheme more flexible. Moreover, the new free parameters may help to eliminate the terms of Hamiltonian which are hard to be realized practically. Furthermore, owing to suitable choice of boundary conditions for parameters, by making a curve fitting, the complex Rabi frequencies Ω_1_ and Ω_2_ of laser pulses can be respective superseded by Rabi frequencies 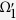 and 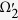 expressed by the superpositions of the Gaussian or trigonometric functions, which can be realized with current experimental technology. The example given in Sec. III has shown that the present scheme can design a Hamiltonian to realize the population transfer for a Rydberg atom successfully and the numerical simulation has shown that the scheme is fast and robustness against the operational imperfection and the decoherence mechanisms. Therefore, the present scheme may be used to construct a Hamiltonian which can be realized in experiments.

## Additional Information

**How to cite this article**: Kang, Y.-H. *et al*. Reverse engineering of a Hamiltonian by designing the evolution operators. *Sci. Rep.* **6**, 30151; doi: 10.1038/srep30151 (2016).

## Figures and Tables

**Table 1:** δΩ′_1_/Ω′_1_ and δΩ′_2_/Ω′_2_ with corresponding fidelity *F*.

δΩ′_1_/Ω′_1_	δΩ′_2_/Ω′_2_	*F*
10%	10%	0.9835
10%	0	0.9951
0	10%	0.9916
0	0	1.0000
−10%	0	0.9938
0	−10%	0.9902
−10%	−10%	0.9822
10%	−10%	0.9875
−10%	10%	0.9887

**Table 2:** δΩ′_1_/Ω′_1_ and *δT*/*T* with corresponding fidelity *F*.

δΩ′_1_/Ω′_1_	*δT*/*T*	*F*
10%	10%	0.9855
10%	0	0.9951
0	10%	0.9942
0	0	1.0000
−10%	0	0.9938
0	−10%	0.9855
−10%	−10%	0.9729
10%	−10%	0.9879
−10%	10%	0.9915

**Table 3:** *δ*Ω′_2_/Ω′_2_ and *δT*/*T* with corresponding fidelity *F*.

δΩ′_2_/Ω′_2_	*δT*/*T*	*F*
10%	10%	0.9688
10%	0	0.9916
0	10%	0.9942
0	0	1.0000
−10%	0	0.9902
0	−10%	0.9855
−10%	−10%	0.9588
10%	−10%	0.9974
−10%	10%	0.9994

**Table 4:** δΩ′_1_/Ω′_1_, δΩ′_2_/Ω′_2_ and *δT*/*T* with corresponding fidelity *F*.

δΩ′_1_/Ω′_1_	δΩ′_2_/Ω′_2_	*δT*/*T*	*F*
−10%	−10%	−10%	0.9469
10%	−10%	−10%	0.9607
−10%	10%	−10%	0.9853
−10%	−10%	10%	0.9926
10%	10%	−10%	0.9990
10%	−10%	10%	0.9956
−10%	10%	10%	0.9713
10%	10%	10%	0.9531

**Table 5:** Ω_0_ *T* for STIRAP and corresponding fidelity *F*.

Ω_0_*T*	*F*
3.154	0.5538
5	0.6263
10	0.8516
15	0.9604
20	0.9898
25	0.9960
30	0.9992

## References

[b1] Lee J., Paternostro M., Kim M. S. & Bose S. Entanglement reciprocation between qubits and continuous variables. Phys. Rev. Lett. 96, 080501 (2006). 16606160 10.1103/PhysRevLett.96.080501

[b2] Yang C. P. Quantum information transfer with superconducting flux qubits coupled to a resonator. Phys. Rev. A 82, 054303 (2010).

[b3] Amniat-Talab M., Guérin S., Sangouard N. & Jauslin H. R. Atom-photon, atom-atom, and photon-photon entanglement preparation by fractional adiabatic passage. Phys. Rev. A 71, 023805 (2005).

[b4] Saffman M., Walker T. G. & Mølmer K. Quantum information with Rydberg atoms. Rev. Mod. Phys. 82, 2313 (2010).

[b5] Sheng Y. B. & Zhou L. Two-step complete polarization logic Bell-state analysis. Sci. Rep. 5, 13453 (2016). 26307327 10.1038/srep13453 PMC4549687

[b6] Zhou L. & Sheng Y. B. Complete logic Bell-state analysis assisted with photonic Faraday rotation. Phys. Rev. A 92, 042314 (2015).

[b7] Zhou L. & Sheng Y. B. Feasible logic Bell-state analysis with linear optics. Sci. Rep. 6, 20901 (2016). 26877208 10.1038/srep20901 PMC4753447

[b8] Zhou L. & Sheng Y. B. Concurrence measurement for the two-qubit optical and atomic states. Entropy 17, 4293 (2015).

[b9] An N. B., Kim J. & Kim K. Entanglement dynamics of three interacting two-level atoms within a common structured environment. Phys. Rev. A 84, 022329 (2011).

[b10] Man Z. X., An N. B. & Xia Y. J. Non-Markovianity of a two-level system transversally coupled to multiple bosonic reservoirs. Phys. Rev. A 90, 062104 (2014).

[b11] Hua M., Tao M. J. & Deng F. G. Fast universal quantum gates on microwave photons with all-resonance operations in circuit QED. Sci. Rep. 5, 9274 (2015). 25787147 10.1038/srep09274 PMC4365390

[b12] Wei. H. R. & Deng F. G. Scalable quantum computing based on stationary spin qubits in coupled quantum dots inside double-sided optical microcavities. Sci. Rep. 4, 7551 (2014). 25518899 10.1038/srep07551 PMC4269895

[b13] Bergmann K., Theuer H. & Shore B. W. Coherent population transfer among quantum states of atoms and molecules. Rev. Mod. Phys. 70, 1003 (1998).

[b14] Král P., Thanopulos I. & Shapiro M. Coherently controlled adiabatic passage. Rev. Mod. Phys. 79, 53 (2007).

[b15] Vitanov N. V., Halfmann T., Shore B. W. & Bergmann K. Laser-induced pophlation transfer by adiabaatic passage techniques. Annu. Rev. Phys. Chem. 52, 763 (2001). 11326080 10.1146/annurev.physchem.52.1.763

[b16] Møler D., Madsen L. B. & Mømer K. Geometric phase gates based on stimulated Raman adiabatic passage in tripod systems. Phys. Rev. A 75, 062302 (2007).

[b17] Kuklinski J. R., Gaubatz U., Hioe F. T. & Bergmann K. Adiabatic population transfer in a three-level system driven by delayed laser pulses. Phys. Rev. A 40, 6741 (1989). 10.1103/physreva.40.6741 9902079

[b18] Lu M., Xia Y., Shen L. T., Song J. & An N. B. Shortcuts to adiabatic passage for population transfer and maximum entanglement creation between two atoms in a cavity. Phys. Rev. A 89, 012326 (2014).

[b19] Zhang L., Wang C. & Sun C. P. Analysis of quasi-adiabatic dynamic process of a 3-level atom in a quantum cavity. Sci. China-Phys. Mech. Astron. 39, 758 (1996).

[b20] Chen B., Shen Q. H., Fan W. & Xu Y. Long-range adiabatic quantum state transfer through a linear array of quantum dots. Sci. China-Phys. Mech. Astron. 55, 1635 (2012).

[b21] Sun C. P. & Ge M. L. Dynamic Lie algebra structure of quantal system and Berry’s phase factor. Chin. Sci. Bull. 35, 1784 (1990).

[b22] Dou F. Q. & Zheng W. Q. High-fidelity population inversion of two-level system. Chin. Sci. Bull., doi: 10.1360/N972015-01021 (2016).

[b23] Solá I. R., Malinovsky V. S. & Tannor D. J. Optimal pulse sequences for population transfer in multilevel systems. Phys. Rev. A 60, 3081 (1999).

[b24] Sugny D. & Kontz C. Optimal control of a three-level quantum system by laser fields plus von Neumann measurements. Phys. Rev. A 77, 063420 (2008).

[b25] Vasilev G. S., Kuhn A. & Vitanov N. V. Optimum pulse shapes for stimulated Raman adiabatic passage. Phys. Rev. A 80, 013417 (2009).

[b26] Torosov B. T. & Vitanov N. V. Smooth composite pulses for high-fidelity quantum information processing. Phys. Rev. A 83, 053420 (2011).

[b27] Torosov B. T., Guérin S. & Vitanov N. V. High-Fidelity adiabatic passage by composite sequences of chirped pulses. Phys. Rev. Lett. 106, 233001 (2011). 21770500 10.1103/PhysRevLett.106.233001

[b28] Demirplak M. & Rice S. A. Adiabatic Population Transfer with Control Fields. J. Phys. Chem. A 107, 9937 (2003).

[b29] Demirplak M. & Rice S. A. On the consistency, extremal, and global properties of counterdiabatic fields. J. Chem. Phys. 129, 154111 (2008). 19045180 10.1063/1.2992152

[b30] Torrontegui E. . Shortcuts to adiabaticity. Adv. Atom. Mol. Opt. Phys. 62, 117 (2013).

[b31] Berry M. V. Transitionless quantum driving. J. Phys. A 42, 365303 (2009).

[b32] Chen X., Lizuain I., Ruschhaupt A., Guéry-Odelin D. & Muga J. G. Shortcut to Adiabatic Passage in Two- and Three-Level Atoms. Phys. Rev. Lett. 105, 123003 (2010). 20867634 10.1103/PhysRevLett.105.123003

[b33] del Campo A. Shortcuts to Adiabaticity by Counterdiabatic Driving. Phys. Rev. Lett. 111, 100502 (2013). 25166641 10.1103/PhysRevLett.111.100502

[b34] Chen X., Torrontegui E. & Muga J. G. Lewis-Riesenfeld invariants and transitionless quantum driving. Phys. Rev. A 83, 062116 (2011).

[b35] Muga J. G., Chen X., Ruschhaup A. & Guéry-Odelin D. Frictionless dynamics of Bose-Einstein condensates under fast trap variations. J. Phys. B 42, 241001 (2009).

[b36] Chen X. . Fast Optimal Frictionless Atom Cooling in Harmonic Traps: Shortcut to Adiabaticity. Phys. Rev. Lett. 104, 063002 (2010). 20366818 10.1103/PhysRevLett.104.063002

[b37] Chen Y. H., Xia Y., Chen Q. Q. & Song J. Efficient shortcuts to adiabatic passage for fast population transfer in multiparticle systems. Phys. Rev. A 89, 033856 (2014).

[b38] Huang X. B., Chen Y. H. & Wang Z. Fast generation of three-qubit Greenberger-Horne-Zeilinger state based on the Lewis-Riesenfeld invariants in coupled cavities. Sci. Rep. 6, 25707 (2016). 27216575 10.1038/srep25707 PMC4877589

[b39] Sun J., Lu S. F. & Liu F. Speedup in adiabatic evolution based quantum algorithms. Sci. China-Phys. Mech. Astron. 55, 1630 (2012).

[b40] Lewis H. R. & Riesenfeld W. B. An exact quantum theory of the time-dependent harmonic oscillator and of a charged particle in a time-dependent electromagnetic field. J. Math. Phys. 10, 1458 (1969).

[b41] Torrontegui E. . Fast atomic transport without vibrational heating. Phys. Rev. A 83, 013415 (2011).

[b42] Muga J. G., Chen X., Ibáñez S., Lizuain I. & Ruschhaupt A. Transitionless quantum drivings for the harmonic oscillator. J. Phys. B 43, 085509 (2010).

[b43] Torrontegui E. . Fast transitionless expansion of cold atoms in optical Gaussian-beam traps. Phys. Rev. A 85, 033605 (2012).

[b44] Masuda S. & Nakamura K. Acceleration of adiabatic quantum dynamics in electromagnetic fields. Phys. Rev. A 84, 043434 (2011).

[b45] Chen Y. H., Xia Y., Chen Q. Q. & Song J. Fast and noise-resistant implementation of quantum phase gates and creation of quantum entangled states. Phys. Rev. A 91, 012325 (2015).

[b46] Chen X. . Fast Optimal Frictionless Atom Cooling in Harmonic Traps: Shortcut to Adiabaticity. Phys. Rev. Lett. 104, 063002 (2010). 20366818 10.1103/PhysRevLett.104.063002

[b47] Chen X. & Muga J. G. Transient energy excitation in shortcuts to adiabaticity for the time-dependent harmonic oscillator. Phys. Rev. A 82, 053403 (2010).

[b48] Schaff J. F., Capuzzi P., Labeyrie G. & Vignolo P. Shortcuts to adiabaticity for trapped ultracold gases. New J. Phys. 13, 113017 (2011).

[b49] Chen X., Torrontegui E., Stefanatos D., Li J. S. & Muga J. G. Optimal trajectories for efficient atomic transport without final excitation. Phys. Rev. A 84, 043415 (2011).

[b50] Torrontegui E. . Fast transport of Bose-Einstein condensates. New J. Phys. 14, 013031 (2012).

[b51] del Campo A. Frictionless quantum quenches in ultracold gases: A quantum-dynamical microscope. Phys. Rev. A 84, 031606(R) (2011).

[b52] del Campo A. Fast frictionless dynamics as a toolbox for low-dimensional Bose-Einstein condensates. Eur. Phys. Lett. 96, 60005 (2011).

[b53] Ruschhaupt A., Chen X., Alonso D. & Muga J. G. Optimally robust shortcuts to population inversion in two-level quantum systems. New J. Phys. 14, 093040 (2012).

[b54] Schaff J. F., Song X. L., Vignolo P. & Labeyrie G. Fast optimal transition between two equilibrium states. Phys. Rev. A 82, 033430 (2010).

[b55] Schaff J. F., Song X. L., Capuzzi P., Vignolo P. & Labeyrie G. Shortcut to adiabaticity for an interacting Bose-Einstein condensate. Eur. Phys. Lett. 93, 23001 (2011).

[b56] Chen X. & Muga J. G. Engineering of fast population transfer in three-level systems. Phys. Rev. A 86, 033405 (2012).

[b57] Chen Z., Chen Y. H., Xia Y., Song J. & Huang B. H. Fast generation of three-atom singlet state by transitionless quantum driving. Sci. Rep. 6, 22202 (2016). 26931812 10.1038/srep22202 PMC4773874

[b58] Song X. K., Zhang. H., Ai Q, Qiu J. & Deng F. G. Shortcuts to adiabatic holonomic quantum computation in decoherence-free subspace with transitionless quantum driving algorithm. New J. Phys. 18, 023001 (2016).

[b59] Zhang J., Kyaw T. H., Tong D. M., Sjövist E. & Kwek L.C. Fast non-Abelian geometric gates via transitionless quantum driving. Sci. Rep. 5, 18414 (2015). 26687580 10.1038/srep18414 PMC4685308

[b60] Chen Y. H., Xia Y., Song J. & Chen Q. Q. Shortcuts to adiabatic passage for fast generation of Greenberger-Horne-Zeilinger states by transitionless quantum driving. Sci. Rep. 5, 15616 (2016). 26508283 10.1038/srep15616 PMC4623608

[b61] Martnez-Garaot S., Torrontegui E., Chen X. & Muga J. G. Shortcuts to adiabaticity in three-level systems using Lie transforms. Phys. Rev. A 89, 053408 (2014).

[b62] Opatrný T. & Mømer K. Partial suppression of nonadiabatic transitions. New J. Phys. 16, 015025 (2014).

[b63] Saberi H., Opatrny T., Mømer K. & del Campo A. Adiabatic tracking of quantum many-body dynamics. Phys. Rev. A 90, 060301(R) (2014).

[b64] Ibáñez S., Chen X. & Muga J. G. Improving shortcuts to adiabaticity by iterative interaction pictures. Phys. Rev. A 87, 043402 (2013).

[b65] Ibáñez S., Chen X., Torrontegui E., Muga J. G. & Ruschhaupt A. Multiple Schrödinger Pictures and Dynamics in Shortcuts to Adiabaticity. Phys. Rev. Lett. 109, 100403 (2012). 23005267 10.1103/PhysRevLett.109.100403

[b66] Song X. K., Ai Q, Qiu J. & Deng F. G. Physically feasible three-level transitionless quantum driving with multiple Schröodinger dynamics. Phys. Rev. A 93, 052324 (2016).

[b67] Torrontegui E., Martnez-Garaot S. & Muga J. G. Hamiltonian engineering via invariants and dynamical algebra. Phys. Rev. A 89, 043408 (2014).

[b68] Torosov B. T., Valle G. D. & Longhi S. Non-Hermitian shortcut to adiabaticity. Phys. Rev. A 87, 052502 (2013).

[b69] Torosov B. T., Valle G. D. & Longhi S. Non-Hermitian shortcut to stimulated Raman adiabatic passage. Phys. Rev. A 89, 063412 (2014).

[b70] Chen Y. H., Wu Q. C., Huang B. H., Xia Y. & Song J. Method for constructing shortcuts to adiabaticity by a substitute of counterdiabatic driving terms. Phys. Rev. A 93, 052109 (2016).

